# *Escherichia coli* Heat-Labile Enterotoxin B Limits T Cells Activation by Promoting Immature Dendritic Cells and Enhancing Regulatory T Cell Function

**DOI:** 10.3389/fimmu.2017.00560

**Published:** 2017-05-15

**Authors:** Alexandre Bignon, Alan P. Watt, Michelle A. Linterman

**Affiliations:** ^1^Lymphocyte Signalling and Development, Babraham Institute, Babraham Research Campus, Cambridge, UK; ^2^Xenovium Limited, Chesterford Research Park, Little Chesterford, UK

**Keywords:** EtxB, immunomodulation, lung, dendritic cells, regulatory T cells

## Abstract

Treatments to limit T cell activation are essential for managing autoimmune and inflammatory disorders. The B subunit of *Escherichia coli* heat-labile enterotoxin (EtxB) is known to ameliorate inflammatory disease *in vivo* but the mechanism by which this is mediated is not well understood. Here, we show that following intranasal administration, EtxB acts on two key cellular regulators of T cell activation: regulatory T cells and dendritic cells (DCs). EtxB enhances the proliferation of lung regulatory T cells and doubles their suppressive function, likely through an increase in expression of the Treg effector molecule CTLA-4. EtxB supports the generation of interleukin-10-producing DCs that are unable to activate T cells. These data show, for the first time, that mucosal EtxB treatment limits T cells activation by acting jointly on two distinct types of immune cells.

## Introduction

Robust immunological tolerance is essential to prevent development of autoimmune and autoinflammatory disorders. The challenge of the immune system is to balance potent effector mechanisms against foreign pathogens, while remaining unresponsive to self-antigens. The mucosal immune system faces a more complex challenge in that it must also regulate commensal microbial communities that are essential for health, in addition to discriminating between self- and non-self-antigens (1, 2). This balance is maintained by a number of cell types, including Foxp3^+^ regulatory T (Treg) cells and immature or tolerogenic dendritic cells (DCs) (3, 4). Treg cells are a population of suppressive CD4^+^ T cells that act to limit the effector functions of other leukocytes such as CD4^+^ T cells, thereby limiting inflammation and tissue damage. Within the mucosa, Treg cells modulate inflammatory responses by producing high levels of interleukin-10 (IL-10), restraining the generation of inflammatory disease (5–7). Tolerogenic DCs are antigen-presenting cells that are thought to able to promote T cell tolerance to a defined antigen. Typically, tolerogenic DCs have an “immature” phenotype, expressing lower levels of the cell surface receptors that are required to activate T cells (8). In the lung, DCs remain in an immature state and fail to activate naïve T cells, unless they receive an activating signal in parallel with antigen (9). However, whether a specific tolerogenic DC lineage exists *in vivo* or whether this is simply a modification of their activation status is unclear; because of this we will refer to these cells as immature DCs (10). Previous studies show that immature DCs may regulate immunological tolerance through different mechanisms such as the induction of T cell anergy, generation of Treg cells and *via* production of IL-10 and transforming growth factor (TGF)-β (4, 11–13). In addition, immature DCs can support Treg cell differentiation *in vivo* through presentation of low levels of antigen in major histocompatibility complex (MHC)-II (14–16). Therapeutic strategies that augment numbers and/or function of Treg cells, immature DCs, or both, represent a way to enhance mucosal tolerance by limiting T cell activation.

The *Escherichia coli* heat-labile enterotoxin is a hetero-oligomeric AB_5_ toxin composed of a toxic enzymatic A subunit and five identical non-toxic B subunits (EtxB) (17). In the context of infection, the B subunit mediates cellular entry of the A subunit into the cytoplasm by binding to GM1 ganglioside receptor, which is ubiquitously expressed by all somatic cells (18). Several studies have demonstrated the immunomodulatory effects of EtxB, with emphasis on its adjuvant properties, although the mechanism by which EtxB act as an adjuvant is not yet known (19–22). Previous studies reported that recombinant EtxB is non-toxic and its effects are dependant on EtxB binding to cell surface receptors, as evidenced by a failure of a non-receptor-binding mutant, EtxB (G33D), to induce any immunomodulatory effects (23–27). At the cellular level, one study suggests that EtxB binding to GM1 receptor induces both caspase-dependent and -independent cell death pathway in CD8^+^ T cells (28). Conversely, a different study highlighted that receptor occupancy by EtxB on B cells is associated with maintenance of B-cell survival by activation of molecules essential for B-cell differentiation (29). Interestingly, binding of EtxB to GM1 receptor seems to be essential for EtxB-mediated antigen presentation by a immortalized murine bone marrow-derived dendritic cell (BMDC) line; however, EtxB did not induce maturation of BMDC (30, 31). At the molecular level, one study reported that receptor binding by EtxB triggers MAPK/ERK kinase activation in B cells (27). However, the precise molecular mechanisms by which EtxB induces direct or indirect effects on immune cells are largely unknown, in particular on DCs and Treg cells. Nevertheless, mucosal administration of EtxB ameliorates the disease severity of type 1 diabetes and collagen-induced arthritis in mice (23, 25). Treatment of these autoimmune mouse models with EtxB administration has been recapitulated by the transfer of splenocytes from EtxB-treated mice. Interestingly, when these splenocytes were devoid of CD4^+^ T cells they could not mediate tolerance, suggesting a role for EtxB in modulating suppressive Treg cells. In support of this model, intranasal (i.n.) administration of EtxB increased the frequency of Foxp3^+^ cells within the CD4^+^ T cell population (24, 26). Together, these studies suggest that EtxB supports tolerance through increasing Treg cell number. However, the mechanism by which EtxB does this has not been determined, nor is it known if EtxB can alter the suppressive capacity of Treg cells.

In addition, i.n. administration of EtxB induces IL-10 and TGF-β1 production by both epithelial cells in nasal-associated lymphoid tissue and CD11b^+^ cells in the cervical lymph nodes which suggest that EtxB may promote a tolerogenic environment (26). *In vitro* EtxB treatment increases viability of DCs and results in lower expression of MHC class II, CD80, and CD86 features of an immature phenotype (32). This suggests that in addition to enhancing Treg cell proportion, EtxB may also promote immature DCs *in vivo*, although this has not yet been demonstrated.

Interestingly, oral, nasal, or sublingual coadministration of the related non-toxic B subunit protein of cholera toxin (CTB) with selected antigens have been found to induce tolerance, including in autoimmune disorders and allergies in several animal models (33). One of the suggested mechanisms is that the coadministration of CTB with antigen results in increase of antigen-specific Foxp3^+^ Treg cells (33). In addition, CTB diminishes the responsiveness of macrophages and monocytes to lipopolysaccharide (LPS) (34).

Our study aimed to determine the mechanism by which EtxB treatment promotes the accumulation of Treg cells at mucosal sites and whether EtxB treatment affects Treg cell function and DC activation status and function. We confirm that i.n. EtxB treatment increases the proportion of IL-10^+^ Foxp3^+^ Treg cells and, for the first time, show that this treatment increased the suppressive function of Treg cells, likely through increases in expression of the key Treg effector molecule CTLA-4 (cytotoxic T-lymphocyte-associated protein 4). In addition, mucosal administration of EtxB also increases the frequency of CD8^−^ cDCs with an immature phenotype and enhances their ability to produce IL-10. We show *in vitro* that EtxB directly promotes immature phenotype in BMDCs that fail to activate naïve CD4^+^ T cells. Together, these data demonstrate that EtxB alters the cellular composition of the lung, promoting a regulatory environment that is likely the cause of the anti-inflammatory activity of this protein.

## Materials and Methods

### Experimental Animals

C57BL/6, TCR7 (35), and ITIB mice (36) (provided by H. Bouabe and K. Okkenhaug) were housed under specific pathogen-free conditions at the Biological Support Unit, Babraham Research Campus, Cambridge, UK. All experiments were approved by the UK Home Office under the UK Home Office license PPL 80/2526, in line with the Scientific Procedures Act (1986).

### Administration of EtxB

EtxB-endotoxin free (Trident Pharmaceuticals, USA) was administered by the intranasal route under inhaled isoflurane anesthesia. Each mouse was administered 100 µg EtxB (as previously described), or heat-inactivated EtxB (95°C for 10 min), in 20 µl of sterile PBS or 20 µl of sterile PBS alone, on three consecutive days (23). The dose of 100 µg EtxB used i.n. has previously been shown to promote tolerance (23). Mice were euthanized at different time points after the last treatment, as indicated in the figure legend.

### Flow Cytometry and Cell Sorting

Single-cell suspensions were prepared from mouse spleen by sieving and gentle pipetting through Falcon 70 µm nylon mesh filters (BD Biosciences, San Jose, CA, USA). To prepare cell suspensions from mediastinal lymph node (mLN), the tissue was incubated with 1 mg/ml Collagenase D (Roche Diagnostics, Mannheim, Germany), and 400 U/ml DNase I (Sigma-Aldrich, St. Louis, MO, USA) for 20 min at room temperature, followed by gentle pipetting to disrupt tissue. Lung lymphocytes were isolated by finely mincing the lung tissues and digesting with 2 mg/ml Collagenase (Sigma-Aldrich, St. Louis, MO, USA) and 0.2 mg/ml DNase I (Roche Diagnostics) at 37°C for 30 min, followed by sieving and gentle pipetting through Falcon 70 µm nylon mesh filters (BD Biosciences). Red blood cells were removed by using ammonium chloride lysis buffer. Cells were washed with PBS with 2% FCS, (PBS–FCS 2%) then stained with antibody cocktails. Different gating strategies were used to define myeloid and common DC subsets as previously described (32) (Figure S2 in Supplementary Material). For intracellular staining, cell suspensions were fixed and permeabilized using the Intracellular Fixation and Permeabilization Buffer Set as per the manufacturer’s instructions (eBioscience, San Diego, CA, USA). Annexin V and DAPI staining procedure was performed following *Annexin V Staining Protocol* from BD Biosciences. To assess IL-10 production *ex vivo*, we used IL-10-β-lactamase reporter mice (ITIB) (36) as described previously. Briefly, IL-10 was detected in cells from ITIB mice using CCF4-AM staining solution supplemented with probenecid, prepared according to the manufacturer’s instructions (Thermo Scientific, Wilmington, DE, USA), and incubated for 75 min at room temperature. Cells were washed with PBS–FCS 2% and analyzed by flow cytometry. For cell sorting, conventional naïve CD4^+^ T cells (B220^−^CD3^+^CD4^+^CD25^−^CD62L^+^) or regulatory T cells (B220^−^CD3^+^CD4^+^CD25^+^) were sorted from the spleen or the lung, respectively, after cell surface staining. FACS was performed with a FACSAria cell sorter (BD Biosciences) and sorted populations were between 95 and 99% purity. Analyses were carried out on an LSR Fortessa (BD Biosciences) using FlowJo software (TreeStar, Ashand, OR, USA).

### Antibodies and Dyes for Flow Cytometry

Antibodies for flow cytometry were from eBioscience except where otherwise indicated: anti-CD4 (Biolegend, London, UK, RM4-5), anti-CD8 (Becton Dickinson, 53-6.7), anti-Foxp3 (FJK-16S), anti-Ki-67 (SolA15), anti-CTLA-4 (UC10-4B01), anti-CD304 (3DS304M), anti-CD25 (PC61.5), anti-CD80 (16-10A1), anti-CD86 (GL1), anti-CD11b (M1/70), anti-CD11c (N418), anti-MHC-II (M5/114.15.2), anti-CD69 (H1.2F3), Annexin V, and DAPI.

### Regulatory T Cell Suppression Assay

Regulatory T cell suppression assay was performed as previously published (37). Briefly, conventional splenic CD4^+^ T cells were isolated from untreated C57BL/6 mice and labeled with 10 µM CellTrace Violet (eBioscience). Conventional CD4^+^ T cells (2.5 × 10^4^/well) were activated with anti-CD3 anti-CD28 coated beads (Gibco by Life Technologies, AR, Oslo) and cultured either alone or with lung CD4^+^CD25^+^ regulatory T cells at a range of ratios from 1:1 to 64:1 (CD4^+^CD25^−^: CD4^+^CD25^+^). After 4 days of culture, proliferation of conventional CD4^+^ T cells was determined by CellTrace violet dilution by flow cytometry and the percentage of suppression of CD4^+^CD25^+^ T cells can be calculated using the following formula: [(% of proliferation of CD4^+^CD25^−^ cells alone − % of proliferation of CD4^+^CD25^−^ treated with CD4^+^CD25^+^)/% of proliferation of CD4^+^CD25^−^ cells alone] as previously described (37).

### BMDC Culture and Treatment

Bone marrow cells were obtained from the femur and tibia of untreated C57BL/6 mice (38). To generate BMDCs, the cells were cultured in RPMI-1640 medium supplemented with 10% FCS, 20 ng/ml of GM-CSF (R&D systems, Abingdon, UK), 10 ng/ml of IL-4 (R&D systems) and 50 µM of 2-Mercaptoethanol (Sigma-Aldrich) for 6 days. To induce activation, BMDCs were treated with 1 mg/ml of LPS (Sigma-Aldrich) for three additional days of culture. To generate tolerogenic DC, bone marrow cells were cultured in RPMI-1640 medium supplemented with 10% FCS, 20 ng/ml of GM-CSF, 20 ng/ml of IL-10, 20 ng/ml of TGF-β (R&D systems), and 50 µM of 2-Mercaptoethanol for 9 days. BMDC cells were cultured for 12 h with hEtxB or EtxB (both at 10 µg/ml) at 37°C in 5% CO_2_. The concentration of hEtxB or EtxB used *in vitro* and the time of treatment was consistent with previously published studies (32).

### Antigen-Specific Presentation Assay

Hen egg lysozyme (HEL) or ovalbumin (OVA) (Sigma-Aldrich) were added to BMDCs at 1 mg/ml for 24 h, cells were fixed in 0.75% paraformaldehyde for 30 min on ice. Naïve TCR7 CD4^+^ T cells were isolated using a MagniSort™ Mouse CD4 Naïve T cell Enrichment Kit (eBioscience) according to the manufacturer’s instructions. 9 × 10^4^ BMDCs that were exposed to HEL or OVA were co-cultured with 2.5 × 10^5^ naïve CD4^+^ transgenic T cells in RPMI-1640 containing 5% FCS (Fixed BMDCs:CD4^+^ T cells = 1:3). CD4^+^ T cell activation was assessed by flow cytometry at 5 h post co-culture by membrane expression of CD69.

### Statistics

Data are presented as mean ± SD. Single comparisons were analyzed using the non-parametric Mann–Whitney *U*-test. All statistical analyses were carried out with GraphPad Prism v6 (La Jolla, CA, USA).

## Results

### The Proportion of Foxp3^+^ Regulatory T Cells Increase Following Intranasal EtxB Treatment

**Figure 1:**
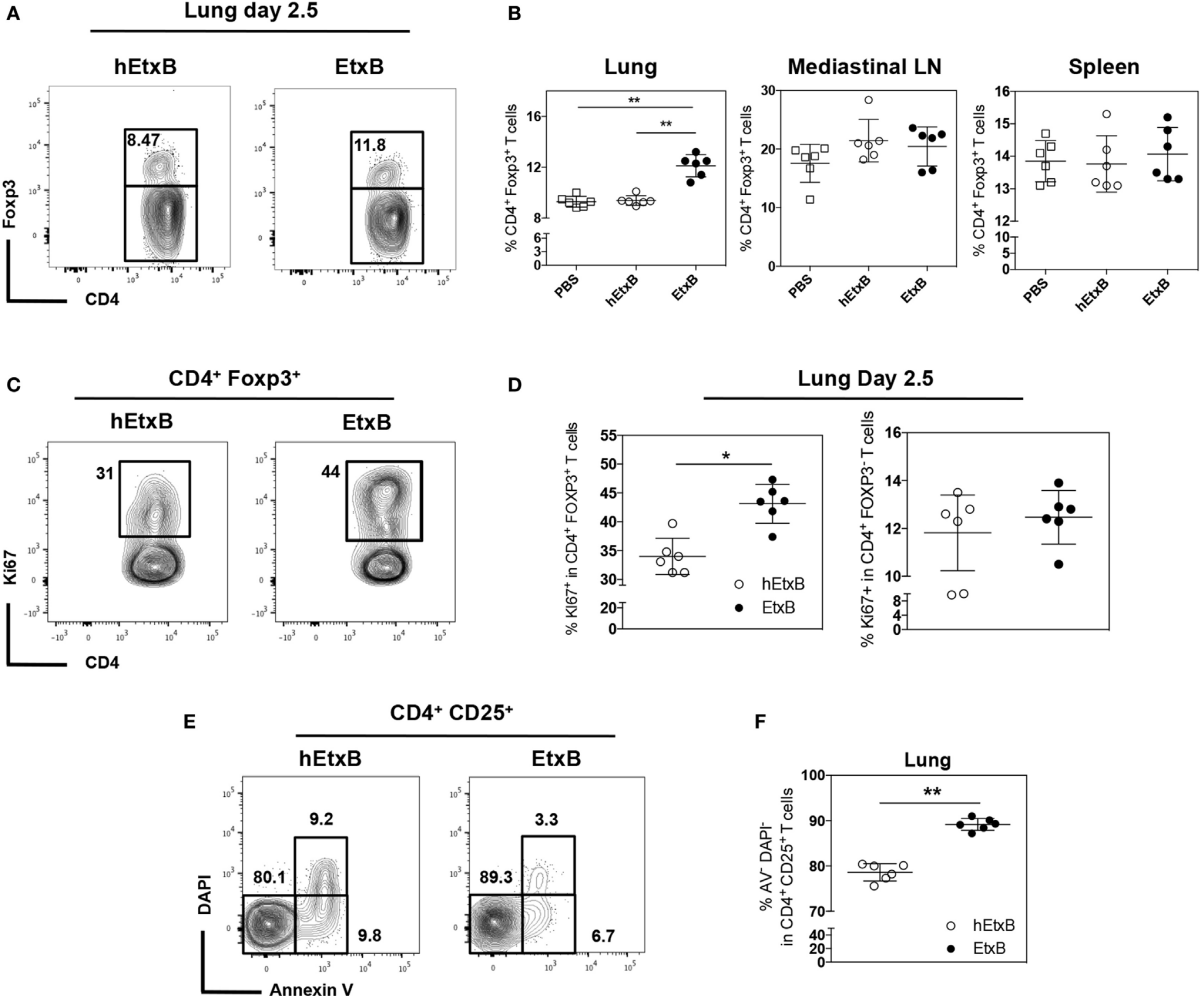
**Intranasal treatment of EtxB increases the proliferation of Treg cells**. C57BL/6 mice were administered, EtxB (100 μg/mouse), heat inactivated EtxB (hEtxB, 100 μg/mouse) or PBS on three consecutive days. On day 2.5 following the last dose, spleen, mediastinal lymph node, and lung were removed and analyzed by flow cytometry. Flow cytometric contour plots **(A)** and dot plots **(B)** show the frequency of Foxp3^+^ regulatory T (Treg) cells in CD4^+^ B220^−^ cells. The frequency of Ki67^+^ proliferating Treg cells or CD4^+^ Foxp3^−^ T cells **(C,D)** and frequency of live (Annexin V^−^ DAPI^−^) CD4^+^ CD25^+^ cells **(E,F)** in the lung. Results are from one experiment representative of three independent experiments, with six mice per group. In dot plots, the mean and SD are represented and each circle represents one mouse: white squares PBS treated animals, white dots hEtxB-treated animals, black dots EtxB-treated animals. *p < 0.05 and **p < 0.005 compared with PBS- or hEtxB-treated mice (Mann–Whitney *U*-test).

Foxp3^+^ Treg cells are powerful mediators of immunological tolerance; their frequency has been reported to increase upon EtxB treatment (24, 26). We first sought to confirm this following i.n. EtxB administration. In the lung, EtxB treatment results in an increased proportion of CD4^+^ T cells expressing Foxp3 2.5 days after treatment (Figures 1A,B) compared to control mice that received PBS or biologically inactive heat-treated EtxB (hEtxB). By contrast, the increased frequency of Treg cells is not detectable 2.5 days posttreatment in the draining mLN or spleen (Figure 1B). Interestingly, an increased proportion of Tregs in the spleen is detectable at day 9 posttreatment (Figure S1A in Supplementary Material), suggesting that the mucosal site of administration is the first and major location of EtxB action on Treg cells. Interestingly, no differences in the spleen, the mLN, or in the lung were observed between PBS- and hEtxB-treated mice at day 2.5 following treatment indicating that hEtxB is a relevant biologically inactive negative control (Figure 1B). While previous studies have reported an increase in Treg cell proportion following EtxB treatment, they have not determined how this occurs. This observation suggests that EtxB may be able to alter Treg cell proliferation and/or survival. To test this, we determined the frequency of proliferating (Ki67^+^) and live (Annexin V^−^DAPI^−^) Treg cells in the lung 2.5 days after EtxB administration. After EtxB treatment, Treg cells had a significantly increased proportion of both Ki67^+^ cells (Figures 1C,D) and live cells (Figures 1E,F). By contrast, EtxB administration is not able to induce the proliferation of CD4^+^ Foxp3^−^ T cells suggesting an EtxB-Treg specific effect and that EtxB alone seems not to be “immunogenic” on naïve/effector CD4^+^ T cells (Figure 1D). Together, the increase in cell proliferation with a decrease in cell death likely accounts for the higher proportion of Treg cells in the lung of EtxB-treated mice and that mucosal sites are the principle location of this effect.

### The Suppressive Function of Treg Cells Is Enhanced by EtxB Treatment

**Figure 2:**
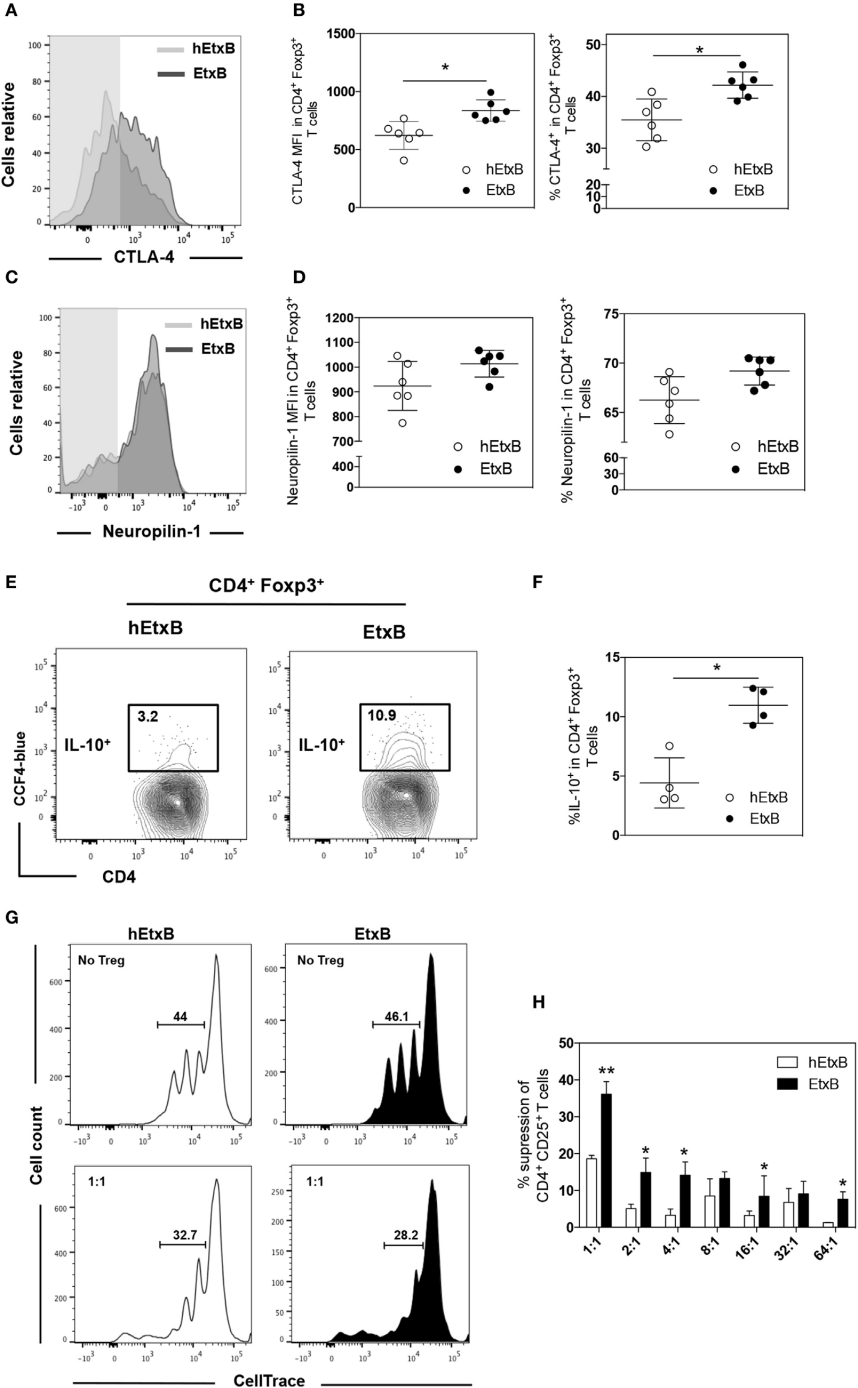
**EtxB increases the suppressive function of Treg cells**. Expression of CTLA-4 **(A,B)** or Neuropilin-1 **(C,D)** on lung B220^−^ CD4^+^ Foxp3^+^ Treg cells from C57BL/6 mice treated intranasally with 100 μg/mouse EtxB or heat-EtxB (hEtxB) for three consecutive days, and analyzed by flow cytometry at 2.5 days after the final treatment. **(E,F)** Intranasal administration of 100 μg/mouse EtxB or hEtxB to ITIB mice. Day 2.5 posttreatment, lungs were removed and analyzed by flow cytometry for the proportion of IL-10^+^ (CCF-4 blue) in B220^−^ CD4^+^ Foxp3^+^ Treg cells. **(G,H)** Groups of 10 C57BL/6 mice were treated intranasally with 100 μg/mouse EtxB or hEtxB for three consecutive days. On day 2.5 posttreatment, lungs were removed and CD4^+^ CD25^+^ Treg cells were flow sorted and were co-cultured with splenic CD4^+^ CD25^−^ T cells isolated from untreated C57BL/6 mice that have been labeled with CellTrace Violet. Cells were cultured at the ratio of CD4^+^CD25^−^: CD4^+^CD25^+^ indicated on the graph. After 4 days, proliferation was determined by CellTrace Violet dilution **(E)** and the percentage of suppression of CD4^+^ CD25^+^ T cells was assessed using the following formula: [(% of proliferation of CD4^+^CD25^−^ cells alone − % of proliferation of CD4^+^CD25^−^ treated with CD4^+^CD25^+^)/% of proliferation of CD4^+^CD25^−^ cells alone] as previously described (37). Results are from one experiment representative of six **(A–D)** or four **(E,F)** mice in each group from three independent experiments. In histograms **(A,C)**, the shaded area represents the fluorescence minus one control. In dot plots, the mean and SD are represented and each circle represents one mouse: white dots hEtxB-treated animals, black dots EtxB-treated animals. Results in **(G,H)** are from one experiment representative of three independent experiments. *p < 0.05 and **p < 0.005 compared with hEtxB-treated mice (Mann–Whitney *U*-test).

Previous work has suggested that EtxB dampens inflammation through increased proportion of Treg cells (23–25), but it is also possible that EtxB functionally alters Treg cells. We sought to determine if mucosal administration of EtxB alters the activation status and/or suppressive capacity of Treg cells. CTLA-4 is a key effector molecule for Treg cell function (39); Treg cells from EtxB-treated mice had increased expression of CTLA-4 compared to control hEtxB-treated mice in the lung, but the expression is unchanged in the mLN (Figures 2A,B and Figures S1B,C in Supplementary Material). In the lung, the expression of Neuropilin-1 on Treg cells is not affected in EtxB-treated mice suggesting no preferentially effect of EtxB on thymic Treg cells or induced peripheral regulatory T cells (Figures 2C,D). Several studies have shown that IL-10 plays a role in Treg cell suppressive function at mucosal sites (40, 41). To investigate the effect of i.n. EtxB administration on IL-10 production by Treg cells in the lung, we used an IL-10-β-lactamase (ITIB) reporter mouse (36). Treatment with EtxB resulted in a twofold increase in the frequency of IL-10-producing Treg cells, compared to control hEtxB-treated mice (Figures 2E,F). Interestingly, EtxB did not alter expression of other markers of Treg induction/activation such as inducible T-cell costimulator, programmed cell death 1, CD101, and CD103 (data not shown). Together, increased CTLA-4 and IL-10 expression suggests that EtxB is able to enhance the functional capacity of Treg cells. To test this hypothesis, we performed an *in vitro* Treg suppression assay (37). CD4^+^CD25^+^ Treg cells were isolated from the lung of EtxB-treated C57BL/6 mice by flow cytometric cell sorting and co-cultured with naïve CD4^+^ T cells from an untreated animal, and the ability of Treg cells to suppress TCR-driven CD4^+^ T cell proliferation was assessed. CD4^+^CD25^+^ Treg cells taken from EtxB-treated mice had twice the suppressive function of Treg cells isolated from hEtxB-treated control animals (Figures 2G,H). These findings demonstrate that mucosal administration of EtxB enhances the suppressive function of Treg cells, describing a novel role for EtxB in modifying Treg cell biology.

### Mucosal EtxB Administration Increases Immature DCs

ExtB has previously been shown to modify innate cells, by reducing the frequency of DC precursors and myeloid precursors in secondary lymphoid tissues (32), and by increasing the expression of IL-10 and TGF*-*β transcripts in CD11b^+^ cells (26). To confirm that EtxB acts on innate cells at mucosal sites, we examined the effect of i.n. EtxB administration on lung dendritic and myeloid cell subsets (Figure S2 in Supplementary Material). Consistent with the previous report (32), EtxB treatment reduced the proportion of conventional DC precursors (pre-cDC, CD11c^low^CD11b^+^CD8^−^MHC-II^−^) and myeloid cell precursors (CD11b^+^CD11c^−^CD8^−^MHC-II^−^) in the lung (Figures S3A,B in Supplementary Material). In addition, we also observed that expression of CD80 was upregulated in plasmacytoid pre-DCs (p-preDC, CD11c^low^CD11b^−^CD8^−^MHC-II^−^), pre-cDC, and myeloid precursors, while CD86 was elevated in pre-cDC in the lung of EtxB-treated mice (Figures S3C,D in Supplementary Material). In the mLN, EtxB treatment reduced only the proportion of myeloid cell precursors (Figures S4A,B in Supplementary Material) and had no effect on the expression of CD80 or CD86 in dendritic and myeloid cell precursors (Figures S4C,D in Supplementary Material). This suggests that EtxB can alter both the proportion and phenotype of innate immune cells in the lung and mLN.

**Figure 3:**
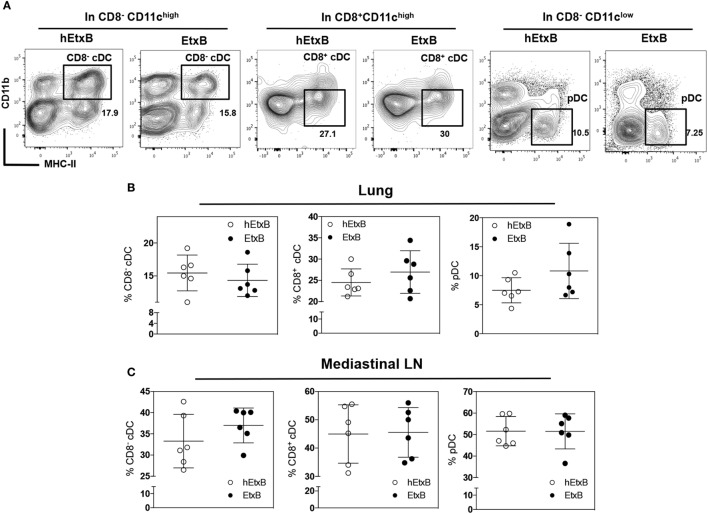
**EtxB does not alter mature dendritic cell (DC) subsets**. C57BL/6 mice were treated intranasally with 100 μg/mouse EtxB or heat-EtxB (hEtxB) for 3 days. On day 2.5 posttreatment, lung and mediastinal lymph node (mLN) were removed and analyzed, flow cytometric contour plots **(A)** and dot plots show the proportion of DCs subsets in the lung **(B)** and mLN **(C)**. Subsets of DC include plasmacytoid DC (pDC, CD11c^low^CD11b^−^CD8^−^MHC-II^+^), CD8^−^ and CD8^+^ conventional DCs (CD8^+^ or CD8^−^ cDC, CD11c^high^CD11b^+^CD8^−/+^ MHC-II^+^). Results are from one experiment representative of six mice in each group from three independent experiments In dot plots, the mean and SD are represented and each circle represents one mouse: white dots hEtxB-treated animals, black dots EtxB-treated animals.

**Figure 4:**
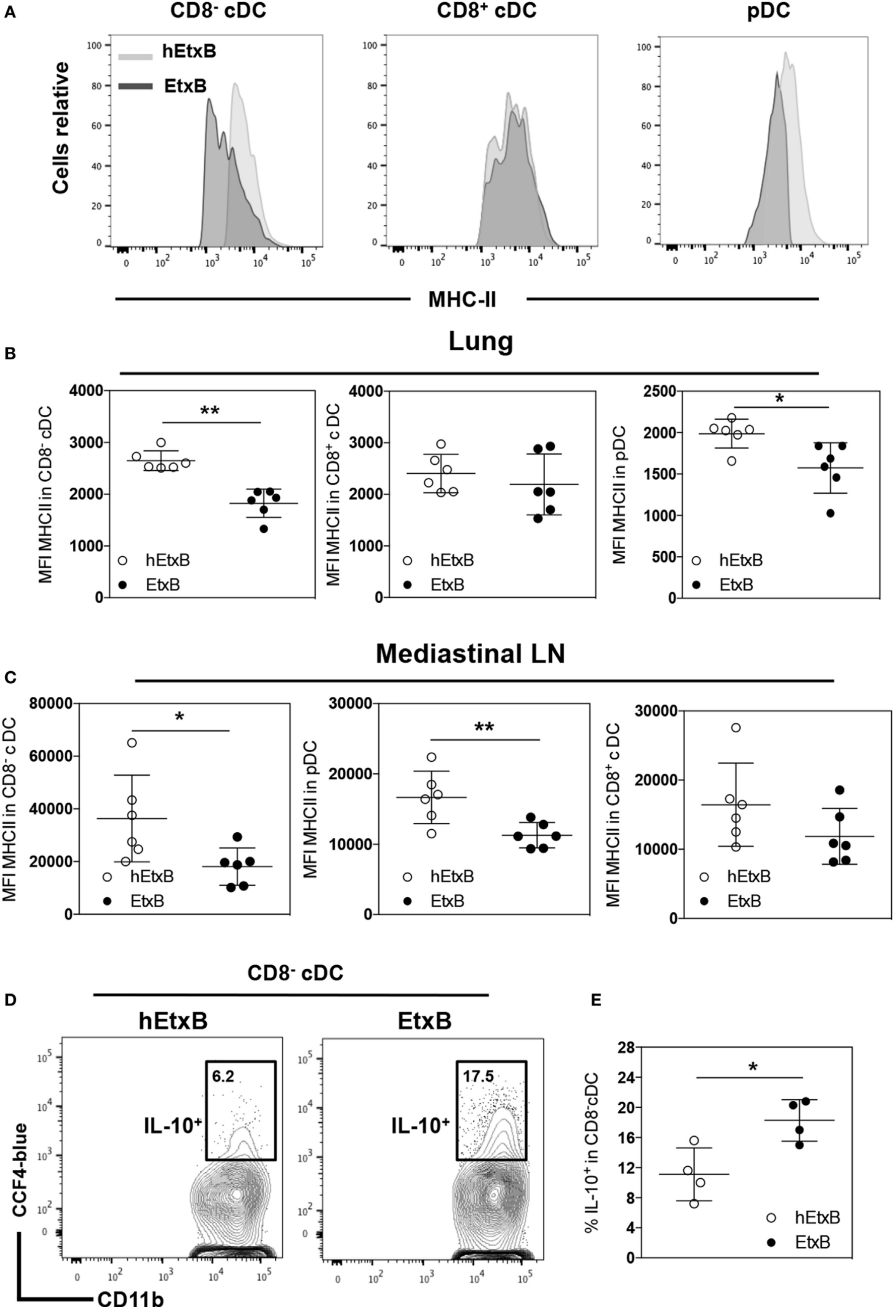
**EtxB promotes immature dendritic cells (DCs)**. C57BL/6 mice were treated intranasally with 100 μg/mouse EtxB or heat-EtxB (hEtxB) for 3 days. On day 2.5 posttreatment, lung and mediastinal lymph node (mLN) were removed and analyzed. Flow cytometric histograms **(A)** and dot plots of membrane expression of major histocompatibility complex (MHC) class II on plasmacytoid DC (pDC, CD11c^low^CD11b^−^CD8^−^MHC-II^+^), CD8^−^, and CD8^+^ conventional DCs (CD8^+^ or CD8^−^ cDC, CD11c^high^CD11b^+^CD8^−/+^ MHC-II^+^) in the lung **(B)** and mLN **(C)**. ITIB mice were administered either 100 μg/mouse EtxB or hEtxB for 3 days. Flow cytometric contour plots **(D)** and dot plots **(E)** represent the frequencies of IL-10^+^ (CCF4-blue) CD8^−^ cDC cells in the lung 1 day posttreatment. In panel **(A–C)**, results are from one experiment representative of six C57BL/6 mice per group from three independent experiments. In **(D,E)**, results are from one experiment representative of four ITIB per group from two independent experiments. In dot plots, the mean and SD are represented and each circle represents one mouse: white dots hEtxB-treated animals, black dots EtxB-treated animals. *p < 0.05 and **p < 0.005 compared with hEtxB-treated mice (Mann–Whitney *U*-test).

Next, we investigated the effect of i.n. EtxB administration on mature DC subsets; plasmacytoid DC (pDC, CD11c^low^CD11b^−^CD8^−^MHC-II^+^), CD8^−^, and CD8^+^ cDCs (CD11c^high^CD11b^+^CD8^−/+^MHC-II^+^) in the lung and in the mLN. The proportions of these DC subsets were not affected by i.n. EtxB administration in either organ (Figures 3A–C). But, we observed that CD8^−^cDC and pDC from EtxB-treated mice have significantly reduced expression of MHC class II, consistent with a profile of “immature” DCs (Figures 4A–C). The low levels of MHC class II on DCs from EtxB-treated mice is intriguing in the context of immunological tolerance, as DCs of this phenotype have also been described to have a poor capacity to activate T cells. Interestingly, the inability of immature DCs to activate T cells is partially dependent on IL-10 production by immature DCs (13, 16, 42). To investigate the ability of EtxB to promote IL-10 production by these immature phenotype DCs, we treated ITIB mice with EtxB or hEtxB as a control. EtxB-treated mice had a significantly increased percentage of IL-10^+^ CD8^−^cDC compared to hEtxB-treated control mice (Figures 4D,E). We did not observe any effect of EtxB treatment on IL-10 expression by the other subsets of DCs or myeloid cells (data not shown). These results show that mucosal administration of EtxB increases the proportion of “immature” IL-10^+^ CD8^−^ cDCs, a new immunoregulatory mechanism by which EtxB can modify the innate immune system at the mucosal surface.

### EtxB Induces Immature BMDCs That Are Unable to Activate T Cells *In Vitro*

**Figure 5:**
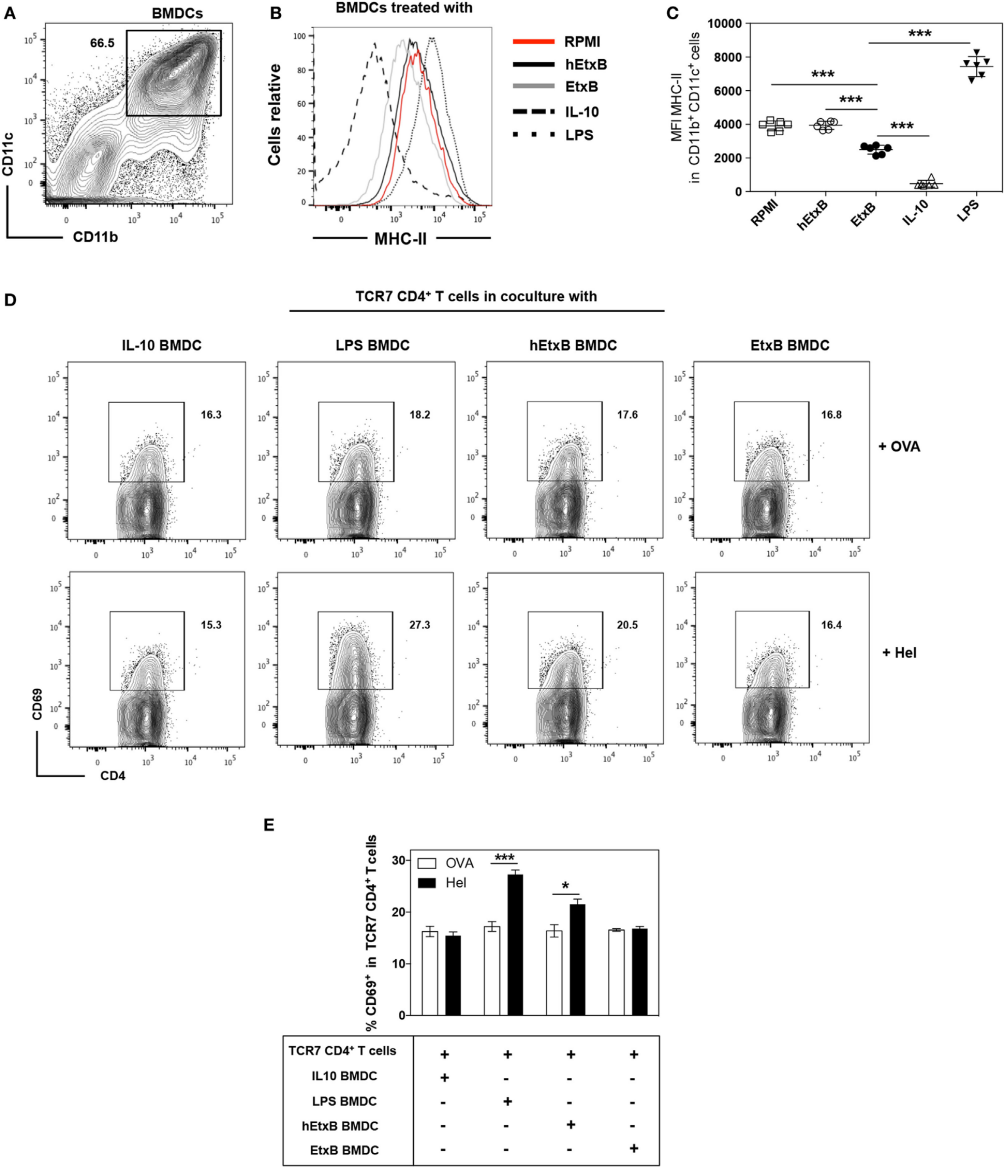
**EtxB inhibits the T cell activation capacity of bone marrow-derived dendritic cells (BMDCs) *in vitro***. BMDCs were induced by culturing bone marrow cells of C57BL/6 mice cultured with GM-CSF, IL-4 and 2-Mercaptoethanol for 6 days. **(A)** Flow cytometric contour plot of CD11b/CD11c expression on BMDC cultures. **(B,C)** Membrane expression of major histocompatibility complex (MHC) class II on BMDC cultures from different conditions as indicated, BMDCs were defined as CD11b^+^ CD11c^+^ cells. **(D)** Flow cytometric contour plots and bar chart **(E)** of CD69 expression on naïve TCR7 CD4^+^ T cells co-cultured with BMDCs generated under the indicated conditions, pulsed with either hen egg lysozyme (HEL) or ovalbumin (OVA) protein and then fixed. In dot plots, the mean and SD are represented and each symbol represents a technical replicate: white squares RPMI only treated cells, white circles hEtxB-treated cells, black circles EtxB-treated cells, white triangles interleukin-10 (IL-10)-induced tolerogenic BMDCs, black triangle lipopolysaccharide (LPS) treated BMDCs. In the bar chart, the height of the bar represents the mean; the error bars the SD. Results are from one experiment representative of three independent experiments. *p < 0.05 and ***p < 0.0005 (Mann–Whitney *U*-test).

These data prompt the hypothesis that EtxB treatment results in an increase of immature DC population that fail to induce T cell activation, which may partly explain the regulatory effects of EtxB *in vivo*. To test this, we assessed the effect of EtxB on *in vitro* generated CD11b^+^ CD11c^+^ BMDCs. Bone marrow cells from C57BL/6 mice were cultured with GM-CSF and IL-4 (38) for 6 days prior to treatment with either RPMI alone or with hEtxB or EtxB followed by analysis of MHC class II expression (Figure 5A). As a positive control, we treated BMDCs with LPS to induce activation, leading to MHC-II upregulation. As a negative control, we generated BMDCs in tolerogenic culture conditions (with IL-10 and TGF-β), which reduced expression of MHC class II (38). EtxB treatment resulted in BMDCs that expressed a lower level of MHC class II compared to control cells. EtxB-treated BMDCs phenotypically resembled *in vitro*-induced immature BMDCs (Figures 5B,C). This suggests that EtxB can act directly on BMDCs to reduce expression of MHC class II and directly promotes an immature state thereby impeding their capacity to activate T cells. To test this, we performed an *in vitro* T cell activation assay with naive TCR7 transgenic CD4^+^ T cells specific for the subdominant H-2^b^ epitope of HEL. LPS-, IL-10-, hEtxB-, or EtxB-treated BMDCs were exposed to 1 mg/ml of HEL, or OVA antigen as negative control, for 24 h. The cells were washed, fixed, and added at a ratio 1:3 to TCR7 cells. Fixation was performed to ensure that the BMDCs did not mature during culture with T cells (43). Incubation of BMDCs with HEL or OVA had no effect on MHC class II, CD80 and CD86 membrane expression (data not shown). As expected, in all conditions, BMDCs exposed to OVA antigen were unable to induce expression of the early activation marker, CD69, on TCR7 CD4^+^ T cells (Figures 5D,E). BMDCs pulsed with HEL and activated with LPS were able to induce CD69 expression on T cells, while IL-10-induced BMDCs were not (Figures 5D,E). We found that HEL-pulsed hEtxB-BMDCs stimulated CD69 expression on transgenic T cells, but that BMDCs treated with EtxB were unable to induce T cell expression of CD69 above background levels (Figures 5D,E). Overall, these findings indicate that EtxB directly promotes *in vitro* “immature” BMDCs that fail to activate naïve CD4^+^ T cells.

## Discussion

EtxB has been shown to ameliorate immune pathology in numerous mouse models of inflammatory disease (23–25). However, the way EtxB alters the immune system to mediate these effects has not been fully elucidated. Here, we show that EtxB acts directly at mucosal sites to increase the proportion and function of suppressive Treg cells and to promote CD8^−^ cDCs with an immature phenotype. Importantly, EtxB is able to act directly on BMDCs to promote immature antigen-presenting cells that do not have the capacity to activate naïve CD4^+^ T cells. Together, these data indicate that the immunoregulatory effects of EtxB work by increasing the frequency of leukocytes that promote tolerance, and further modulating their function to dampen T cell activation.

Our results build substantially on previous studies indicating that i.n. administration of EtxB increased the frequency of Foxp3^+^ Treg cells (24, 26). In our study, we confirm that EtxB increases Treg proportion and show that this occurs rapidly and preferentially in mucosal tissues. The data show that the likely cause of increased Treg cell frequency following EtxB treatment is due to increased proliferation of thymic-derived Treg cells, with a concomitant decrease in cell death. While the molecular mechanism behind this cellular phenotype is unknown, studies on other cell types may provide some insight into the potential cause. It has been reported that EtxB–receptor interaction on B cells leads to induction of phosphatidylinositol-3 kinase (PI3-kinase)-dependent signaling cascades that regulate B cell activation (27). Because of the central role of PI3-kinase in cell growth, differentiation, survival and proliferation in T cells, and important role in Treg cell function (44), one can speculate that a mechanism dependent on PI3-kinase activation could contribute to increase proliferation and survival of Treg cells driven by EtxB.

Our results demonstrate that mucosal administration of EtxB promotes Tregs cells with a greater suppressive capacity. There are numerous mechanisms used by Tregs cell to impart suppression, including production of inhibitory cytokines (TGF-β, IL-10, IL-35), inhibitory receptors (CTLA-4, lymphocyte-activation gene 3), cytotoxicity (Granzyme/Perforin) and metabolic disruption (IL-2 deprivation-mediated apoptosis) (45). Blockade of CTLA-4 results in autoimmune disease and colitis in normal mice (46, 47), exacerbates diabetes in diabetes-prone non-obese mice (48), and abrogates Treg cell-mediated suppression (47, 49). Although, previous works suggested that the Treg cells that develop in absence of CTLA-4 have acquired compensatory suppressive mechanisms through enhanced TGF-β- or IL-10-dependent pathways (49, 50), one study revealed a non-redundant role for CTLA-4 expression by Treg cells to limit lymphopenia-induced CD4 T-cell expansion *in vivo*, which seems to be independent of IL-10, IL-35, TGF-β, or IDO (51). However, despite the important role of CTLA-4 for suppressive capacity, IL-10 is also important for Treg function, particularly at mucosal surfaces (52). Here, we observe that EtxB administration increases expression of IL-10 by Treg cells and we show that EtxB treatment results in increased in CTLA-4 expression. This results in enhanced functional capacity of Treg cells from EtxB-treated mice.

It has been previously demonstrated that EtxB treatment alters the innate immune system by reducing the proportion of precursors of cDCs and myeloid cells (32). Our work confirms this result in the lung and further shows that surface expression of the costimulatory ligand CD80 is upregulated on these cells. DCs are sentinels of the immune system and play an essential role in the maintenance of immune tolerance (14, 53). The potential of DCs to induce regulatory responses could be directly related to their maturation status (54). T cell inactivation in the lung can be induced by immature DCs that express low surface levels of MHC class II and costimulatory ligands. Notably, immature DCs are characterized by increased expression of programmed death-ligand 1, decreased expression of MHC class II, and decreased expression of costimulatory molecules (such as CD86 or CD40) (55–57). These “immature” DCs have the capacity to induce or expand Tregs cells (14). We observed that lung CD8^−^cDC and pDC have significantly reduced membrane expression of MHC class II after EtxB treatment, consistent with an immature phenotype. In addition, we show that EtxB-treated mice had a significantly increased percentage of IL-10^+^ CD8^−^cDC in the lung compared to hEtxB-treated mice. Taken together, this suggests that following mucosal EtxB administration, the DCs with an immature phenotype are promoted; further, these cells produce IL-10 locally that may play a role in the establishment of an immunoregulatory microenvironment in the lung. In the mLN, EtxB treatment has no effect on Treg cells and DCs precursors at day 2.5 following i.n. administration. However, we observed increase proportion of immature CD8^−^cDC and pDC. Interestingly, although expression of CCR7 is considered an indicator of activated DCs, some “immature” DCs in peripheral tissues such as the lung can also up regulate CCR7, which allows them to migrate to the secondary lymphoid organs (58).

In this study, we have addressed the question of how administration of EtxB induces an immunoregulatory microenvironment in the lung. Our results suggest that EtxB is able to promote Treg cells and “immature” DCs. In both cases, there is a marked increase in IL-10 production. Interestingly, IL-10 is able to support the induction of both immature DCs and Treg cells (14, 59). Previous studies have shown increased expression of *Il10* transcript in epithelial cells and CD11b*^+^* cells following EtxB treatment (25, 26). Taken together, IL-10 production after EtxB administration is likely one of the key mechanisms supporting the increase in Treg cells and immature DCs. Interestingly, ERK1/2 pathway is one of the signaling cascades that is activated in macrophages and DCs that results in IL-10 expression (60). Of note, Polumuri et al shown that TLR4 engagement in murine innate cells activates the PI3-kinase/Akt pathway and promotes IL-10 production that is reversed by PI3-kinase inhibition (61). Because EtxB induces PI3-kinase and MAPK/ERK kinase signaling cascades in B cells, it would be interesting to assess the potential link between theses pathways and IL-10 production in Treg and DCs following EtxB administration. Also, how EtxB directly or indirectly promotes Treg cells and immature DCs able to produce IL-10 warrants further study.

Taken together, our study demonstrates that EtxB exerts its effects *in vivo* mainly at mucosal surfaces. It limits T cell activation through two mechanisms: first through increasing “immature” IL-10^+^ DCs that cannot activate T cells and second through increasing the proportion and function of Treg cells that limit T cell expansion. This model of EtxB action could explain why mucosal administration of EtxB protects from different T cell-dependent autoimmune diseases (23, 25) and suggests mucosal administration of EtxB as attractive therapeutic treatment for inflammatory disorders.

## Ethics Statement

All experiments were performed according to the regulations of the UK Home Office Scientific Procedures Act (1986) under the UK Home Office license PPL 80/2526.

## Author Contributions

AB conceived, designed the study, and performed the experiments and wrote the manuscript. AW contributed to study design and reviewed the manuscript. ML conceived and designed the study and wrote the manuscript.

## Conflict of Interest Statement

The authors declare that this study received funding from Trident Pharmaceuticals. The funder was not involved in the study design or collection, analysis, or interpretation of the data.
