# Supplementary material for: Escherichia coli Heat-Labile Enterotoxin B Limits T Cells Activation by Promoting Immature Dendritic Cells and Enhancing Regulatory T Cell Function
Source: Front Immunol. 2017 May 15;8:560. doi: 10.3389/fimmu.2017.00560 (PMC5430108; doi:10.3389/fimmu.2017.00560)
Supplement: Supplementary file 1 [file presentation_1.pdf]

Supplementary Material

Escherichia coli heat-labile enterotoxin B limits T cells activation by promoting immature dendritic cells and enhancing regulatory T cell function

Alexandre Bignon[*]**, Alan P. Watt and Michelle A. Linterman**[*]

*** Correspondence:** Alexandre Bignon, PhD, (Email: alexandre.bignon@babraham.ac.uk) and Michelle Linterman, PhD, (Email: michelle.linterman@babraham.ac.uk)

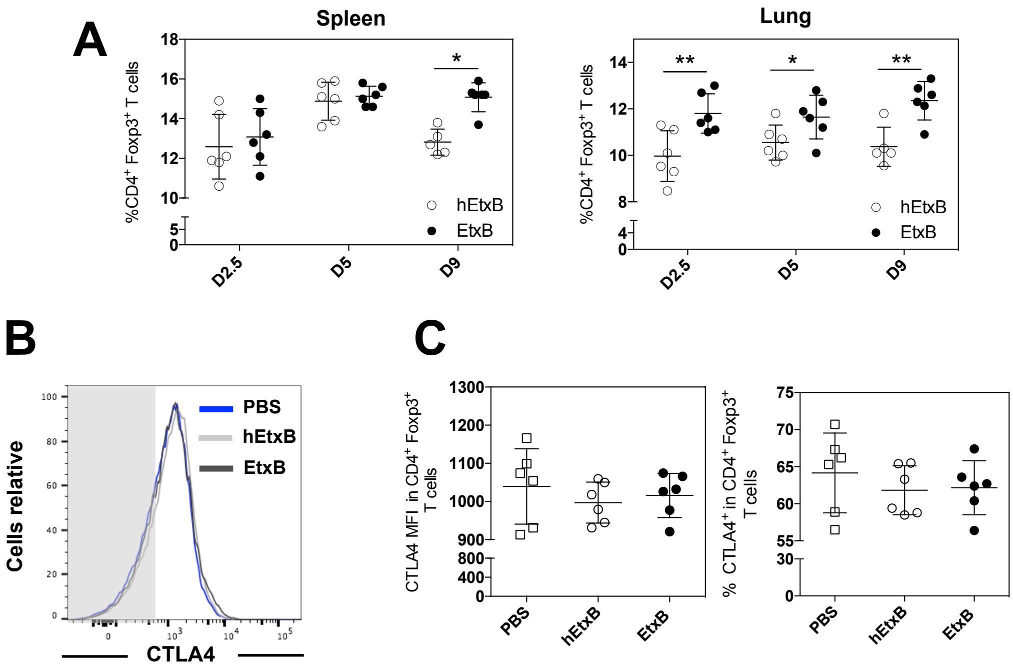

<u>Supplementary Figure 1.</u> Effects of Etxb administration on regulatory T cells. Groups of six C57BL/6 mice were administered, EtxB (100 µg/mouse), heat inactivated-EtxB (hEtxB) or PBS on three consecutive days. On day 2.5, 5 and 9 spleen and lung were isolated and analysed by flow cytometry for the proportion of B220^{-} CD4^{+} Foxp3^{+} regulatory T cells (A). Expression of CTLA-4 (B and C) on mediastinal lymph node of B220^{-} CD4^{+} Foxp3^{+} Treg cells from groups of C57BL/6 mice treated intranasally with 100 µg/mouse EtxB, hEtxB or PBS for three consecutive days, and analyzed by flow cytometry at 2.5 days after the final treatment. Results are from one experiment representive of six mice per group from three independent experiments. In histogram (B), the shaded area represents the fluorescence minus one control. (A and C) In dot plots, the mean and SD are represented and each circle represents one mouse: white squares PBS treated animals, white dots hEtxB treated animals, black dots EtxB treated animals. *p < 0.05 and **p < 0.005 compared with hEtxB-treated mice (as determined using the Mann-Whitney U-test).

Supplementary Figure 2 Bignon et *al.*

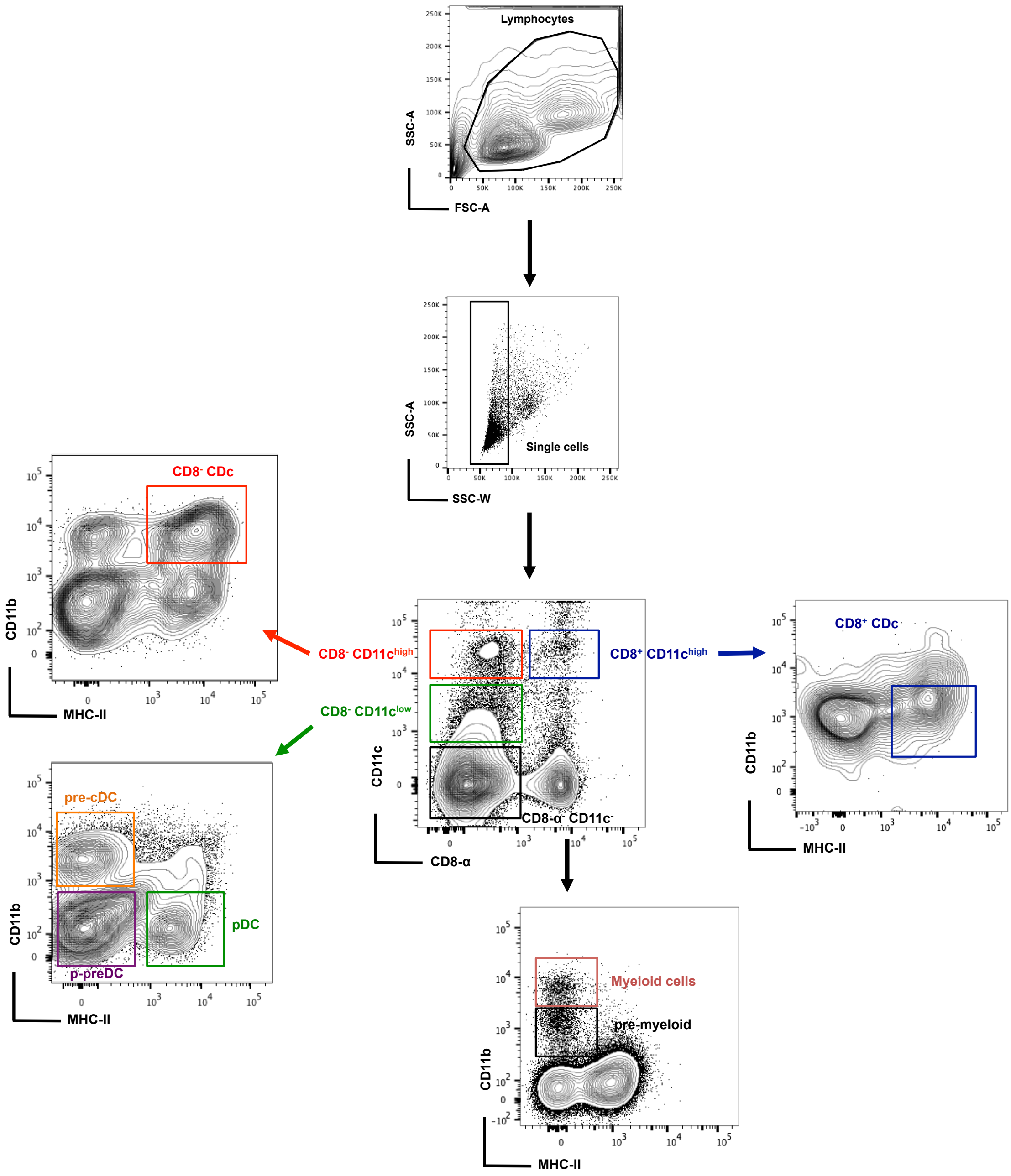

<u>**Supplementary Figure 2.**</u> **Gating strategy for identification of dendritic cells and myeloid cells subsets.** Groups of six C57BL/6 or four ITIB mice were administered, EtxB (100 µg/mouse), heat inactivated-EtxB (hEtxB) or PBS on three consecutive days. On day 1 or 2.5 lung or mediastinal lymph node were removed and analyzed by flow cytometry. Plots from one representative experiment showing dendritic and myeloid cells in the lung are shown. Cell debris was gated out using FSC versus SSC. Different gating strategies were used to define myeloid and common dendritic cells subsets on the basis of CD11c, CD11b, CD8 and MHC class II expression. Subsets are defined as: conventional DC precursors (pre-cDC, $CD11c^{low}CD11b^{+}CD8^{-}MHC\text{-}II^{-}$), myeloid cell precursors (pre-myeloid, $CD11b^{+}CD11c^{-}CD8^{-}MHC\text{-}II^{-}$), plasmacytoid pre-dendritic cells (p-preDC, $CD11c^{low}CD11b^{-}CD8^{-}MHC\text{-}II^{-}$), plasmacytoid DC (pDC, $CD11c^{low}CD11b^{-}CD8^{-}MHC\text{-}II^{+}$), $CD8^{-}$ and $CD8^{+}$ conventional DCs ($CD8^{+}$ or $CD8^{-}$ cDC, $CD11c^{high}CD11b^{+}CD8^{-}$ or $^{+}$ $MHC\text{-}II^{+}$) and myeloid cells ($CD11b^{-}CD11c^{-}CD8^{-}MHC\text{-}II^{-}$).

Supplementary Figure 3 Bignon *et al.*

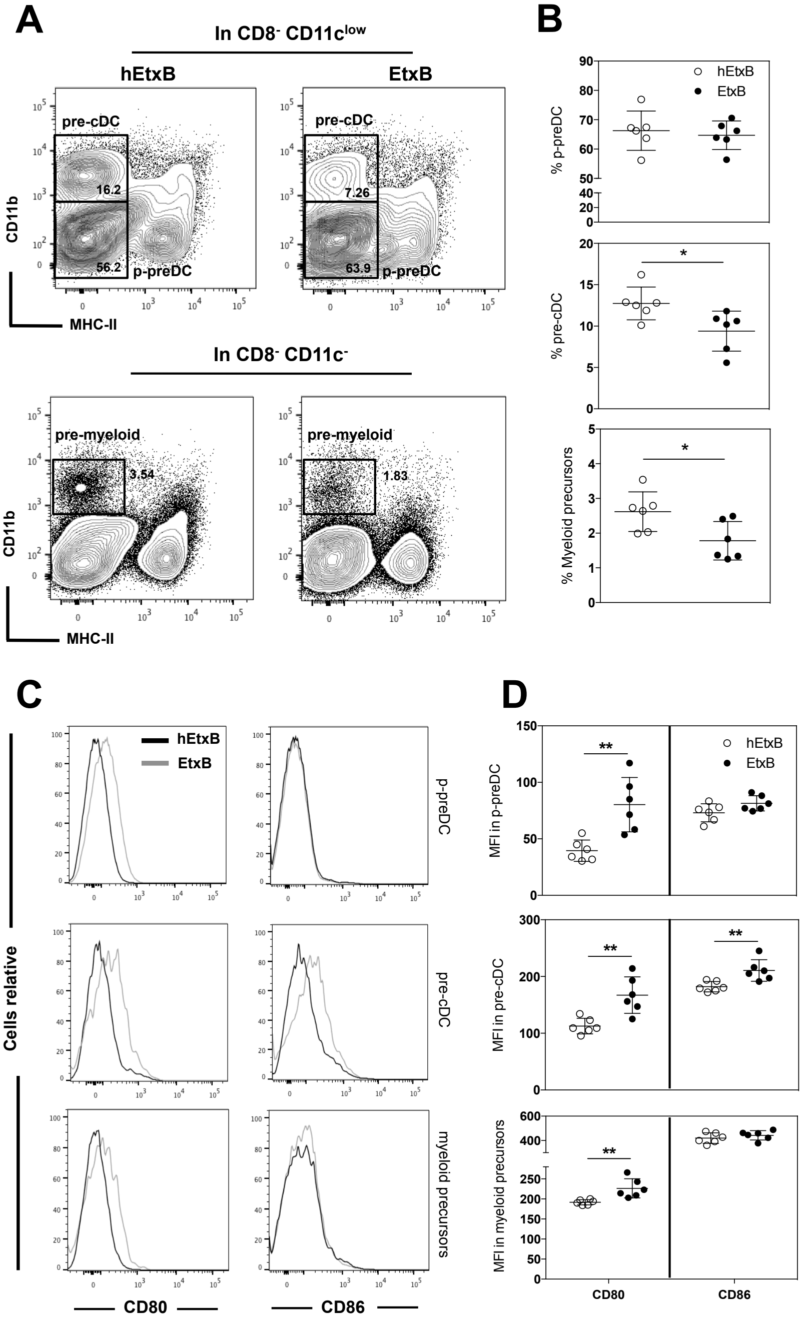

<u>Supplementary Figure 3</u>. EtxB reduces the proportion of dendritic and myeloid precursors cells in the lung. Flow cytometric contour plots (A) and dot plots (B) of C57BL/6 mice treated intranasally with 100 µg/mouse EtxB or heat-EtxB (hEtxB) for three consecutive days. On day 2.5 post treatment, lung was removed and analyzed by flow cytometry for the proportion of plasmacytoid predendritic cells (p-preDC,$CD11c^{low}CD11b^{-}CD8^{-}MHC\text{-}II^{-}$), precursors of conventional DCs (pre-cDC, $CD11c^{low}CD11b^{+}CD8^{-}MHC\text{-}II^{-}$) and myeloid precursors (pre-myeloid, $CD11b^{+}CD11c^{-}CD8^{-}MHC\text{-}II^{-}$). Flow cytometric histograms (C) and dot plots (D) of membrane expression of CD80 and CD86 gated on p-preDC, pre-cDC and myeloid precursors in the lung of hEtxB or EtxB treated mice. In dot plots, the mean and SD are represented and each circle represents one mouse: white dots hEtxB treated animals, black dots EtxB treated animals. Results are from one experiment representative of six mice per group from three independent experiments. $*p < 0.05$ and $**p < 0.005$ compared with hEtxB-treated mice (as determined using the Mann-Whitney U-test).

Supplementary Figure 4 Bignon et *al.*

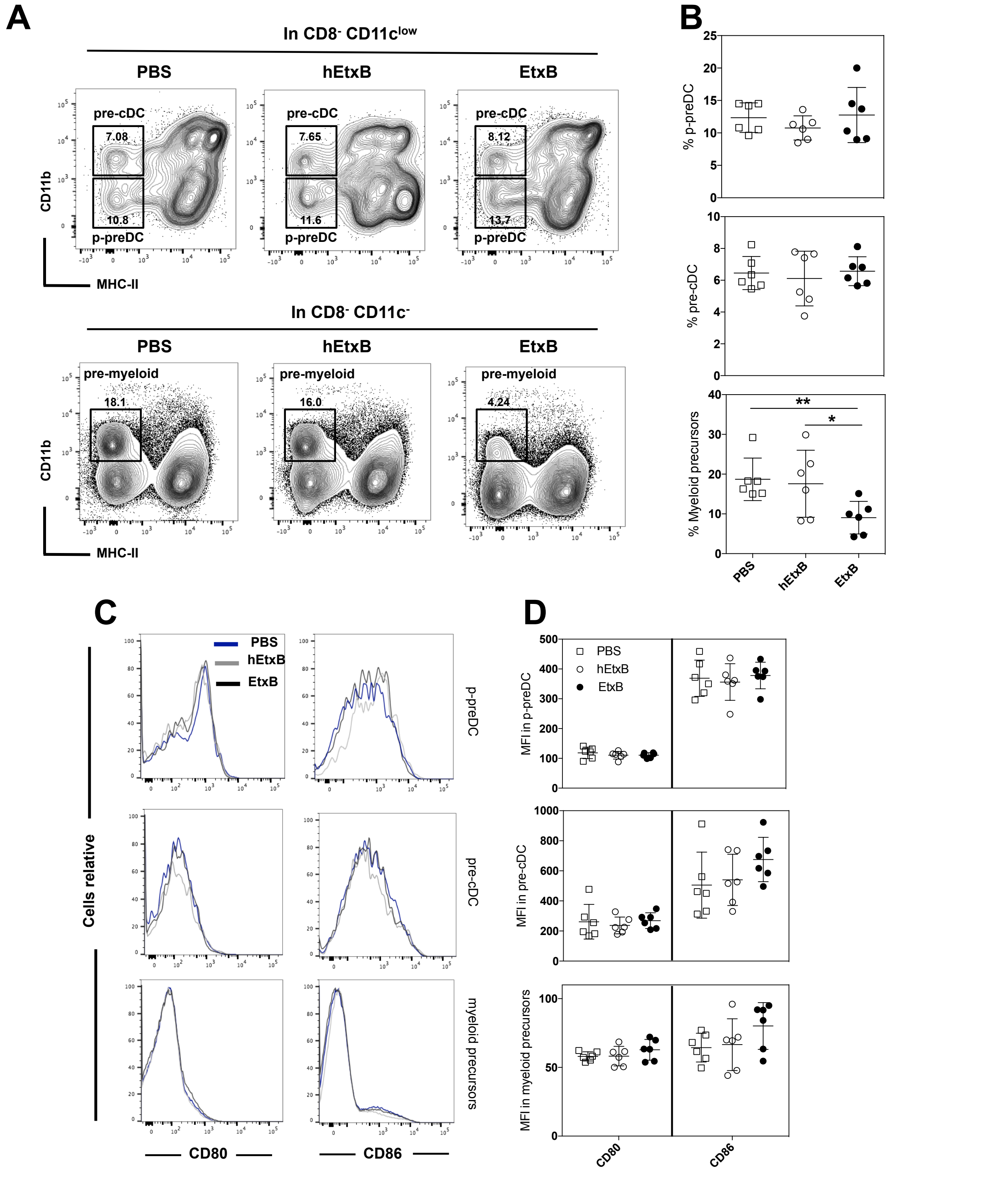

<u>Supplementary Figure 4.</u> EtxB reduces the proportion of myeloid precursors cells in the mediastinal lymph node. Flow cytometric contour plots (A) and dot plots (B) of C57BL/6 mice treated intranasally with 100 µg/mouse EtxB, heat-EtxB (hEtxB) or PBS for three consecutive days. On day 2.5 post treatment, mediastinal lymph nodes were removed and analyzed by flow cytometry for the proportion of plasmacytoid predendritic cells (p-preDC, $CD11c^{low}CD11b^{-}CD8^{-}MHC\text{-}II^{-}$), precursors of conventional DCs (pre-cDC, $CD11c^{low}CD11b^{+}CD8^{-}MHC\text{-}II^{-}$) and myeloid precursors (pre-myeloid, $CD11b^{+}CD11c^{-}CD8^{-}MHC\text{-}II^{-}$). Flow cytometric histograms (C) and dot plots (D) of membrane expression of CD80 and CD86 gated on p-preDC, pre-cDC and myeloid precursors in the lung of PBS, hEtxB or EtxB treated mice. In dot plots, the mean and SD are represented and each circle represents one mouse: white squares PBS treated animals, white dots hEtxB treated animals, black dots EtxB treated animals. Results are from one experiment representative of six mice per group from three independent experiments. *$p < 0.05$ and **$p < 0.005$ compared with PBS- or hEtxB-treated mice (as determined using the Mann-Whitney U-test).
